# The life-course impact of smoking on hypertension, myocardial infarction and respiratory diseases

**DOI:** 10.1038/s41598-017-04552-5

**Published:** 2017-06-28

**Authors:** Kaiye Gao, Xin Shi, Wenbin Wang

**Affiliations:** 1 Donglinks School of Economics and Management, University of Science and Technology Beijing, Beijing, China; 2 School of Mathematics, University of Manchester, Manchester, UK; 3 Business School, Manchester Metropolitan University, Manchester, UK

## Abstract

The objective of this study was to examine the impact of smoking on respiratory diseases, hypertension and myocardial infarction, with a particular focus from a life-course perspective. In this study, 28,577 males from a Chinese longitudinal survey were analysed. The effects of smoking on the risk of respiratory diseases, hypertension and myocardial infarction were assessed from a life-course perspective and a current view separately. No significant associations were found between smoking and the risk of incident respiratory diseases, hypertension and myocardial infarction in the group younger than 35. Among study participants aged between 36–55 and 56–80, smoking was positively associated with the risk of incident respiratory diseases, hypertension and myocardial infarction from the life-course perspective, and the risk increased with age. In contrast, the results from a current view showed inverse associations between smoking and the risk of the diseases mentioned above. Our findings highlight that it is essential to quantify the effects of smoking from a life-course perspective in future research and to suggest that smokers quit smoking as soon as possible, regardless of the temporary side effects of quitting.

## Introduction

Smoking can affect the risk of incident hypertension^1, 2^ and myocardial infarction^3, 4^. As chronic diseases, smoking-caused hypertension and myocardial infarction can not only reduce life expectancy^5, 6^ but also detrimentally affect quality of life^7, 8^. We should note that tobacco smoking is also one of the main preventable causes of hypertension and myocardial infarction^9, 10^, which means that these diseases could be controlled to a large extent by smoking cessation. Abundant evidence has proven that cigarette smoking is associated with some symptoms of the respiratory system^11, 12^.

Although smoking has been identified as a health risk factor, some studies documented opposite findings in terms of hypertension and myocardial infarction. For instance, some researchers have reported lower blood pressure levels among smokers compared to former smokers^13^ and increases in blood pressure after smoking cessation^14, 15^. The results from a male steel workers’ follow-up have revealed that the rate of hypertension among continuous smokers is lower than never-smokers and ex-smokers^16^. Smoking has also been primarily associated with a decreased risk of pregnancy-induced hypertension among growth-restricted babies^17^. As a result, it is still unclear to what extent cigarette smoking is a risk factor for the development of hypertension^18^. Similar phenomena were found for myocardial infarction. One study found that those patients who quit smoking after myocardial infarction generally had a somewhat more severe clinical course than those who continued to smoke^19^. Moreover, related studies suggested that there is no significant dose-response relationship between smoking and the risk of hypertension and myocardial infarction^20, 21^.

In the studies mentioned above, most only used current smoking behaviour (status classification: non-smokers, current smokers and former smokers; cigarette consumption classification: light smokers, heavy smokers and so on) to analyse the effects of smoking on health. For instance, Green^13^ and Okubo^20^ classified smokers by current smoking status and number of cigarettes smoked per day in their study, while Peltier^17^ only used the data for smoking in a specific period. This may lead to a biased evaluation of the effects of smoking. There are several possible causes accounting for this problem. First, smoking generally has a long-term, cumulative effect on health, so current smoking may not damage health immediately and smokers recover slowly from the damage caused by smoking^22–25^. Specifically, the initiation of chronic diseases is also an agonizingly slow process, so current smoking cannot be a direct indicator of these diseases. Second, smoking can produce a temporary excitement leading to an optimistic self-estimation of health status, and continual smoking can further mask the early warning symptoms of some diseases^26^. Last, since smokers may change their smoking habit at times, current smoking status or cigarette consumption may not effectively reflect tobacco intake^27, 28^. Given the current evidence and research, the impact of smoking on health, particularly on chronic diseases, may be delayed, and thus, current smoking status or consumption may not be closely correlated with the current health status.

Quitting smoking is usually associated with either increased concern regarding health or willingness to improve health status^29^. However, the contradictory effects of current smoking consumption on hypertension and myocardial infarction, as well as the insignificant dose-response relationship between them, have become strong resistances for those smokers who want to quit smoking. Therefore, it remains critical to study the effects of smoking on hypertension and myocardial infarction using information from a life-course perspective, instead of current smoking status or consumption^30^. Herein, from Chinese longitudinal survey data, we proposed a new variable that can reflect smoking consumption over a life-course range. We first examined the effects of smoking on hypertension and myocardial infarction using the life-course-adjusted number of cigarettes smoked per day (LCS) and the current numbers of cigarettes smoked per day (CCS) separately and compared the results. Second, similar analyses were also conducted for respiratory diseases as a comparison, due to their close relationship with smoking^11, 12^. Finally, some reasonable explanations about the impact of smoking on respiratory diseases, hypertension and myocardial infarction are discussed separately, which can help people to enhance their awareness of quitting smoking and inform them of the specific hazards of smoking, rather than a fuzzy sense of ‘smoking is harmful to health’.

## Results

### Sample population characteristics

As noted, Table 1 illustrates the descriptive characteristics of the population sample, including the number, percentage, and CCS and LCS in different groups classified by age, BMI, educational attainment, occupation types and region. In addition, the patients with respiratory diseases, hypertension, and myocardial infarction among the study samples are also shown in Table 1. Notably, the average CCS was larger than the average LCS in most subgroups, and significant differences in CCS and LCS were observed in almost all subgroups.

**Table 1:** Descriptive Statistics of Sample.

Variable	Number	Per cent %	CCS	p Value	LCS	p Value
All samples	28577	100	9.46	—	5.77	—
Age						
18~35	8120	28.41	8.15	—	3.20	—
36~55	12362	43.26	11.46	—	7.10	—
56~80	8095	28.33	8.06	<0.01	6.45	<0.01
BMI						
Normal	20354	71.23	9.89	—	5.87	—
Over weight	6630	23.20	8.58	—	5.67	—
Obese	1593	5.57	7.68	<0.01	4.94	<0.01
Educational attainment						
Primary or less	11019	38.56	10.07	—	6.60	—
Middle	14267	49.92	9.57	—	5.50	—
College	3291	11.52	4.18	<0.01	6.96	<0.01
Occupation types						
Unemployed	6408	22.42	6.87	—	5.56	—
White collar	3933	13.76	8.93	—	5.09	—
Light physical	4536	15.87	9.72	—	5.73	—
Hard physical	13700	47.94	10.75	<0.01	6.08	<0.01
Region						
East	9430	33.00	8.49	—	5.06	—
Middle	11894	41.62	9.96	—	6.04	—
West	7253	25.38	9.89	<0.01	6.26	<0.01
Drinking behaviour						
Non-drinkers	12857	44.99	7.05	—	4.43	—
Barely	3106	10.87	9.31	—	5.20	—
Less frequently	4442	15.54	10.29	—	5.92	—
Frequently	8172	28.60	12.87	<0.01	8.03	<0.01
Diseases						
Respiratory	621	2.17	8.38	0.02	7.14	<0.01
Hypertension	2069	7.24	7.05	<0.01	6.06	0.05
Myocardial Infarction	227	0.79	5.56	<0.01	5.28	0.40

The p Values came from F tests between subgroups.

### Impact of CCS and LCS

We categorised our study samples into three analysis groups based on age (under 35, 36–55, and 56–80 years old). The results in Table 2 show that among participants less than 35 years of age, no significant (p < 1) association was found between the CCS or LCS and the risk of any disease. However, a noticeable fact was that the significance for CCS (0.1846, 0.1731 and 0.451) in the three age groups was generally stronger than for LCS (0.9105, 0.5984 and 0.7067).

**Table 2:** Statistics for average number of cigarettes smoked per day for those aged under 35 years.

Term	CCS			LCS			
	N	OR	CI	p Value	OR	CI	p Value
Respiratory Diseases	120	0.8571	0.6831–1.0765	0.1846	1.0141	0.7965–1.2914	0.9105
Hypertension	52	1.2238	0.9154–1.6367	0.1731	1.0304	0.7543–1.4065	0.8503
Myocardial Infarction	4	0.8485	0.5531–1.3015	0.4515	0.9191	0.5912–1.4284	0.7067

The unit of CCS and LCS is 10 cigarettes per day.

Table 3 illustrates the results for the participants aged between 36 and 55. In this group, the correlations between LCS and the risk of these three diseases were significant. The OR of respiratory diseases caused by increased LCS was 1.1057 (95% CI: 0.9861, 1.2407, p: 0.0863), while the data for CCS were not significant (p: 0.6386). The LCS had a significant negative correlation with the risk of incident hypertension (OR 0.1.0754, 95% CI 1.0050 to 1.1503) and myocardial infarction (OR 1.2952, 95% CI 1.0202 to 1.6445), whereas CCS was significantly associated with increased risk for these three diseases (OR 0.8800, 95% CI 0.8079 to 0.9578 and OR 0.7916, 95% CI 0.6484 to 0.9965).

**Table 3:** Statistics for average number of cigarettes smoked per day for those aged between 36 and 55 years.

Term	CCS			LCS			
	N	OR	CI	p Value	OR	CI	p Value
Respiratory Diseases	250	0.9714	0.8597–1.0970	0.6386	1.1057’	0.9861–1.2407	0.0863
Hypertension	592	0.8800*	0.8079–0.9578	0.0032	1.0754*	1.0050–1.1503	0.0359
Myocardial Infarction	123	0.7916*	0.6484–0.9665	0.0217	1.2952*	1.0202–1.6445	0.0336

‘*’Indicates p < 0.05. Results that approach significance are indicated with the symbol ‘”. The unit of CCS and LCS is 10 cigarettes per day.

The ORs for the participants aged between 56 and 80 are provided in Table 4. CCS was negatively associated with the risk of developing respiratory diseases, hypertension and myocardial infarction (OR 0.8720, 0.9312, 0.7428). In contrast, the increase in LCS was a significant risk factor for respiratory diseases (OR 1.1245, 95% CI 1.0212 to 1.2370), hypertension (OR 1.1046, 95% CI 1.0161 to 1.2012) and myocardial infarction (OR 1.4202, 95% 1.0356 to 1.8048).

**Table 4:** Statistics for average number of cigarettes smoked per day for those aged between 56 and 80 years.

Term	CCS			LCS			
	N	OR	CI	p Value	OR	CI	p Value
Respiratory Diseases	252	0.8720*	0.7621–0.9980	0.0471	1.1245*	1.0212–1.2370	0.0167
Hypertension	1425	0.9312*	0.8711–0.9960	0.0371	1.1046*	1.0161–1.2012	0.0197
Myocardial Infarction	100	0.7428*	0.5777–0.9549	0.0202	1.4202*	1.0356–1.8048	0.0165

‘*’Indicates p < 0.05. Results that approach significance are indicated with the symbol ‘”. The unit of CCS and LCS is 10 cigarettes per day.

### The OR trends

The LCS-related risk of incident respiratory diseases, hypertension and myocardial infarction increased with age (Tables 2–4). In contrast, CCS showed unclear trends in the risk for these diseases. The ORs for myocardial infarction decreased with age, while respiratory diseases and hypertension showed no significant trend. It was also noticeable that compared with the other two groups, the group aged between 56 and 80 years old had more significant outcomes (Table 4).

## Discussion

In this comparative prospective cohort study, we found that the increase in LCS resulted in a significantly increased risk of developing respiratory diseases, hypertension and myocardial infarction. Initially, the magnitude of this effect may be modest, but it increases with age. This was mainly because the incidences of these diseases and the accumulative effect of smoking increase with age^31^. In addition, the number of significant outcomes in the elderly group was higher than in the young group. Among the participants aged between 56 and 80 years, all of the risks of developing these diseases were found to be significantly associated with CCS, while none of the outcomes were significant among the participants who were under the age of 35. This was probably due to the low incidence of these diseases among young adults.

Although cigarette smoking has many well-established detrimental health effects, there is a debate about the variable effects of smoking on hypertension and myocardial infarction. In particular, smoking cessation decreases inflammation but may bring weight gain^2, 32–35^. Clinical and animal studies have confirmed a strong relationship between obesity and hypertension^36^. Accumulating evidence points to visceral obesity as the most important risk factor for hypertension and cardiovascular disease^37^. In particular, weight gain could cause higher blood pressure, heart rate, and hyperlipidaemia^16, 38^. These may increase the risk of developing hypertension and further lead to a higher risk of myocardial infarction^14, 38–40^. This is consistent with our finding that CCS was inversely associated with the risk of hypertension and myocardial infarction. However, the increase in LCS was associated with higher risks of hypertension and myocardial infarction in our study. This is also consistent with previous research finding that cigarette intake is closely associated with a higher risk of hypertension and myocardial infarction long-term^1–3^.

Similar outcomes were observed in the results for respiratory diseases. However, the explanation for the results of respiratory diseases was different by respiratory disease. There are some subtypes of respiratory diseases that are not chronic diseases, such as acute upper respiratory infection, and these non-chronic respiratory diseases can be cured easily short-term. On the other hand, patients with respiratory diseases usually have a greater and more urgent need to stop smoking than the average smoker^12^. Thus, regarding respiratory diseases, the effects of current smoking status or dose may become unstable due to the fact that many smokers just quit smoking for a short term for curing non-chronic diseases, whereas ex-smokers are usually those with serious chronic diseases. Our results also revealed the variable effects of CCS on the risk of respiratory diseases. However, smoking has long been suggested to damage the respiratory tract^41^. With age, the risk of respiratory diseases becomes increasingly higher among smokers, and many of them have chronic respiratory diseases that are hard to cure. This was also shown in our study, where increases in LCS are associated with a higher risk of respiratory diseases.

LCS reflected the real smoking status and dose more effectively and reasonably than CCS. We observed that LCS had more significant outcomes than CCS in the elderly. We also found that LCS was associated with an elevated risk, while CCS was generally related to a reduced risk. Moreover, the LCS showed an increasing trend with age groups, whereas the data for CCS illustrated an unclear trend. In addition, the comparison between CCS and LCS gave some valid explanations as to why some studies found that smoking can positively affect human health in terms of respiratory diseases, hypertension and myocardial infarction. To our knowledge, this is the first time a continuous variable from a life-course perspective was used to quantify the impact of smoking on risks of respiratory diseases, hypertension and myocardial infarction, and we proved that this is more reasonable than only from a current view.

An important limitation of our study was the limited waves of our surveyed samples (average approximately 3.5 per person). This may lead to a biased estimation of the LCS and further cause a deviation in the outcomes. Another limitation was that our study mainly focused on respiratory diseases, hypertension and myocardial infarction. We did not elucidate the relation between this new smoking indicator variable with other outcomes (e.g., cancers) due to the lack of data. Thus, such assessments would need more research.

In conclusion, our study suggested that life-course-adjusted smoking consumption is significantly positively associated with risks of incident respiratory diseases, hypertension and myocardial infarction, and these risks will further increase with age. In contrast, the effect of current smoking consumption is unclear. Our analyses also gave some reasonable explanations of the insignificance of the “dose-relationship” and the phenomenon of “positive effects”. Concerns should be raised that smoking can damage health over the long-term, although the effects may not appear immediately or at a young age. Temporary clinical symptoms caused by smoking cessation cannot be a resistance for smokers who are trying to quit smoking. Furthermore, it is important that future research about the relationship between smoking and health should be conducted from a life-course perspective.

## Methods

### Study design and population

We used data from the Chinese Health and Nutrition Survey (CHNS), a longitudinal survey conducted by the Carolina Population Centre at the University of North Carolina at Chapel Hill, USA, and the National Institute of Nutrition and Food Safety at the Chinese Centre for Disease Control and Prevention. CHNS used a multistage random cluster method to draw samples from nine provinces that varied substantially in geography, economic development, public resources, and health indicators^42^. A standardized questionnaire was used to collect detailed information for demographic and socioeconomic background, health, and nutrition from households and their members. It also includes collected information on community services and infrastructure from a knowledgeable community resident.

There are 19,000 participants from approximately 4400 households in the CHNS. The study was conducted in 1989, and there were eight survey follow-up waves between 1991 and 2011. For our study, we excluded the 1989 wave because it did not collect information about the smoking behaviour of the respondents. From 1991 onwards, each wave of the survey asked whether the respondent had ever smoked a cigarette or a pipe and how many cigarettes had been smoked per day recently. Due to the difficulty of assigning an underlying disease from clinical symptoms in ageing people, all analyses were restricted to diseases occurring at ages 18–79, with censoring when men reached 80 years old (or moved away from study areas). Females were not included in our study due to a low rate of smoking^43^. Excluding participants who had invalid, error and incomplete cases on smoking variables, the sample size for the analyses was 28,577.

### Ethical statement

Ethics permission was obtained from the Institute for Nutrition and Food Safety, the China Center for Disease Control (China CDC). All participants were fully informed of the purpose of the study and invited to participate voluntarily. Written consent letters were also obtained from each participant. Methods were carried out in accordance with the approved guidelines.

### Independent variables

There are two independent variables in our analysis. The first one is *the current number of cigarettes smoked per day* (CCS). This variable was directly collected from the questionnaire. We assigned a value of “0” to CCS for never smokers and ex-smokers. The other variable is *the life-course-adjusted number of cigarettes smoked per day* (LCS), which was derived from the CHNS data. The LCS was calculated by dividing the total number of cigarettes smoked (until the participant was surveyed) by the survival time from birth (day); see Equation (1). In other words, LCS is the average number of cigarettes smoked per day from the life-course perspective.

1 $$LC{S}_{n}=\frac{({t}_{r}-{t}_{s})\times {C}_{r}\times \frac{1}{2}+{\sum }_{k=r}^{n}({t}_{k}-{t}_{k-1})\times ({C}_{k}+{C}_{k-1})\times \frac{1}{2}\times I}{{t}_{n}},$$

where

*LCS*_*n*_       LCS at the *n* ^th^ survey

*t*_*n*_           Age of the first survey (the r^th^ survey in total) that participant started to report a smoking status.

*t*_*s*_           Age of starting smoking

*t*_*k*_           Age of the *k* ^th^ survey after the participant started to report a smoking status.

*C*_*r*_         Current number of cigarettes smoked per day at the *r* ^th^ survey that participant started to report a smoking status.

*C*_*k*_         Current number of cigarettes smoked per day at the *k* ^th^ survey that participant started to report a smoking status.

*I*           Indicator of whether in a smoking status (0 if the participant has quit smoking)

Both independent variables were treated as continuous variables. To our best knowledge, self-reported smoking behaviour can be used to measure smoking consumption in subsequent population-based studies^44, 45^.

### Dependent variables

We extracted three (respiratory diseases, hypertension and myocardial infarction) different disease diagnosis outcomes (occurred or not) from the CHNS questionnaire as dependent variables. The respiratory diseases outcome of respondents was obtained from the question, “What was the doctor’s diagnosis of your illness or injury?” and one of the choices, “respiratory disease”. Diagnosis outcomes for hypertension and myocardial infarction were collected at the session of the disease history in the questionnaire. All dependent variables are binary, where “1” represents suffering from this disease and “0” otherwise.

### Covariates

The analyses were adjusted for three groups of covariates. The first group described biological characteristics, including age and body mass index (BMI). Age was included because it is closely correlated with both smoking status and health status. Similarly, BMI was covered because the relationship between smoking and these diseases may be affected by body weight. In this study, we treated age as a continuous variable and stratified our samples by baseline BMI using World Health Organization criteria (normal: <24 kg/m^2^, over-weight: 18.5 to <25 kg/m^2^, and obese: >=30 kg/m^2^)^46^.

In the second group, there are three demographic indicators: region, education and occupation. Region was a three-category classification that is highly correlated with economic development: east — Liaoning, Jiangsu, and Shandong; middle — Heilongjiang, Henan, Hubei, and Hunan; and west — Guangxi and Guizhou. The categories of educational attainment included 0–6 years (primary school or less), 6–12 years (middle school to high school or the equivalent) and ≥13 years (completed a university or other tertiary education). The occupations were classified into three categories: unemployed, white collar (professional, government), light physical labour (skilled worker, service and merchant) and hard physical labour (farmer, factory worker, manufacturing worker and transportation worker).

The third group collected data regarding alcohol consumption behaviour. The frequency of drinking beer or other alcoholic beverages was used to evaluate the drinking status. Participants who answered “almost every day and 3–4 times a week” were coded as a frequent drinker; “once or twice a week” was included as less-frequent drinker; and “once or twice a month” was regarded as barely drinking; “no more than once a month and never drink” was coded as non-drinker.

### Statistical analysis

Descriptive statistics and F tests were applied to different subgroups (Table 1). In longitudinal data analysis, mixed-effects models can impose time independent effects for each entity that are possibly correlated with the dependent variables, so we can use it to remove the unobserved time-invariant heterogeneity^47^. Thus, treating CCS and LCS as risk factors (10/day as the unit used in analyses), CCS and LCS odds ratios (OR) and 95% confidence intervals (CI) were calculated separately for risks of incident of the three types of disease. Tables 2–4 are based on a mixed effects logistic regression model for longitudinal binary response data, and the statistical significance was 2-sided and denoted at *p* < 0.05^48^. All analyses were conducted in three different age groups (18–35, 36–55 and 56–80 years) and were analysed using R version 3.3.2.
